# Developmental exposure to Ethinylestradiol affects transgenerationally sexual behavior and neuroendocrine networks in male mice

**DOI:** 10.1038/srep17457

**Published:** 2015-12-07

**Authors:** Lyes Derouiche, Matthieu Keller, Anne Hélène Duittoz, Delphine Pillon

**Affiliations:** 1 PRC, UMR 7247 INRA/CNRS/Université François-Rabelais de Tours/IFCE, Nouzilly, France

## Abstract

Reproductive behavior and physiology in adulthood are controlled by hypothalamic sexually dimorphic neuronal networks which are organized under hormonal control during development. These organizing effects may be disturbed by endocrine disrupting chemicals (EDCs). To determine whether developmental exposure to Ethinylestradiol (EE2) may alter reproductive parameters in adult male mice and their progeny, Swiss mice (F1 generation) were exposed from prenatal to peripubertal periods to EE2 (0.1–1 μg/kg/d). Sexual behavior and reproductive physiology were evaluated on F1 males and their F2, F3 and F4 progeny. EE2-exposed F1 males and their F2 to F4 progeny exhibited EE2 dose-dependent increased sexual behavior, with reduced latencies of first mount and intromission, and higher frequencies of intromissions with a receptive female. The EE2 1 μg/kg/d exposed animals and their progeny had more calbindin immunoreactive cells in the medial preoptic area, known to be involved in the control of male sexual behavior in rodents. Despite neuroanatomical modifications in the Gonadotropin-Releasing Hormone neuron population of F1 males exposed to both doses of EE2, no major deleterious effects on reproductive physiology were detected. Therefore EE2 exposure during development may induce a hypermasculinization of the brain, illustrating how widespread exposure of animals and humans to EDCs can impact health and behaviors.

Early life is a period of unique sensitivity during which endogenous hormones exert organizational effects on brain structure and function. During critical developmental periods, embryonic sex hormones promote sexual differentiation and cause permanent changes in the architecture of limbic-hypothalamic circuits by specifying cell number, density of dendritic and axonal connections, and neurotransmitter phenotype^1^,^2^. In the male, masculinization and defeminization are the processes whereby the brain is organized to express sex-specific reproductive mating behavior and appropriate hormonal secretion within the hypothalamic-pituitary-gonadal (HPG) axis in adulthood^3^. These processes result from specific neural networks being induced by androgen production during the perinatal period, notably in rodents^4^. Testosterone acts mainly indirectly *via* estrogen receptors (ERs) after its aromatization into estradiol (E2)^5^. The major brain region mediating masculinization is the preoptic area (POA)^6^. In the murine POA, a cluster of calbindin-expressing cells containing more cells in males than in females corresponds to the sexually dimorphic nucleus (SDN) in the rat and is involved in the control of male sexual behavior^7^. The ontogenesis of the hypothalamic sexually dimorphic neuronal networks involved in the control of reproductive behavior and physiology depends on sex hormones, therefore during development the hypothalamus constitutes a key target for endocrine disrupting chemicals (EDCs).

EDCs are natural and synthetic molecules widespread in the environment which interfere with endogenous endocrine processes, and thus alter physiological functions. Pharmaceutical 17α-Ethinylestradiol (EE2) has recently been added to the European Directive 2013/39/EU^8^ of priority substances found in surface water in the European monitoring list, the so-called “watch list”. EE2 is a potent synthetic estrogen widely used in oral contraceptives, hormone replacement therapy and cancer (breast and prostate) treatments^9^. Much more resistant to metabolism and inactivation than E2 in humans^10^, after its excretion EE2 enters the wastewater network and is not completely removed during wastewater treatment processes^11^. Consequently, EE2 is one of the major pharmaceutical products found as a contaminant in effluent waters. It is much more active than E2 because of the properties of the ethinyl group at the C17α position^10^, exhibiting a high estrogenic activity and exerting its biological effects *via* interactions with various ERs through the same mechanisms as E2^9^. Its estrogenic activity is also higher than other estrogenic EDCs such as bisphenol A and phthalates.

Therefore it is reasonable to consider that environmental EE2 contamination could have deleterious effects on animal and human health^12^, even at concentrations as low as ng.L^−1^, constituting a potential risk for animal and human populations. To assess the level of this risk, it needs to be evaluated correctly.

There is now compelling evidence that ontogenesis of hypothalamic networks are altered after EDCs exposure during development^13^,^14^. We have previously demonstrated that exposure to environmentally relevant doses of EE2 from embryonic day 10 to 14 alters the ontogenesis of the Gonadotropin-Releasing Hormone (GnRH) neurons in the mouse embryo by increasing the number of these neurons^15^. To assess whether such developmental effects could impact on reproductive function in adulthood, we designed a transversal study in mice to evaluate the consequences of developmental exposure to low doses of EE2 (0.1 and 1 μg/kg (body weight)/day) on reproductive behavior, physiology and neuroanatomy of adult males. The aim was to determine whether exposure to estrogenic EE2 from embryonic development up to the peripubertal period could induce a hypermasculinization of the hypothalamic neuroendocrine networks, leading to alterations in the reproductive function of adult male mice (F1 generation) and their progenies (F2, F3 and F4 generations). Sexual behavior, plasma testosterone concentrations and genital tract anatomy were evaluated. Then the neuroanatomy of GnRH and kisspeptin neurons, key hypothalamic regulators of reproduction, and the sexually dimorphic calbindin cell population were investigated.

## Results

### Effect of EE2 developmental exposure on sexual behavior

**Figure 1:**
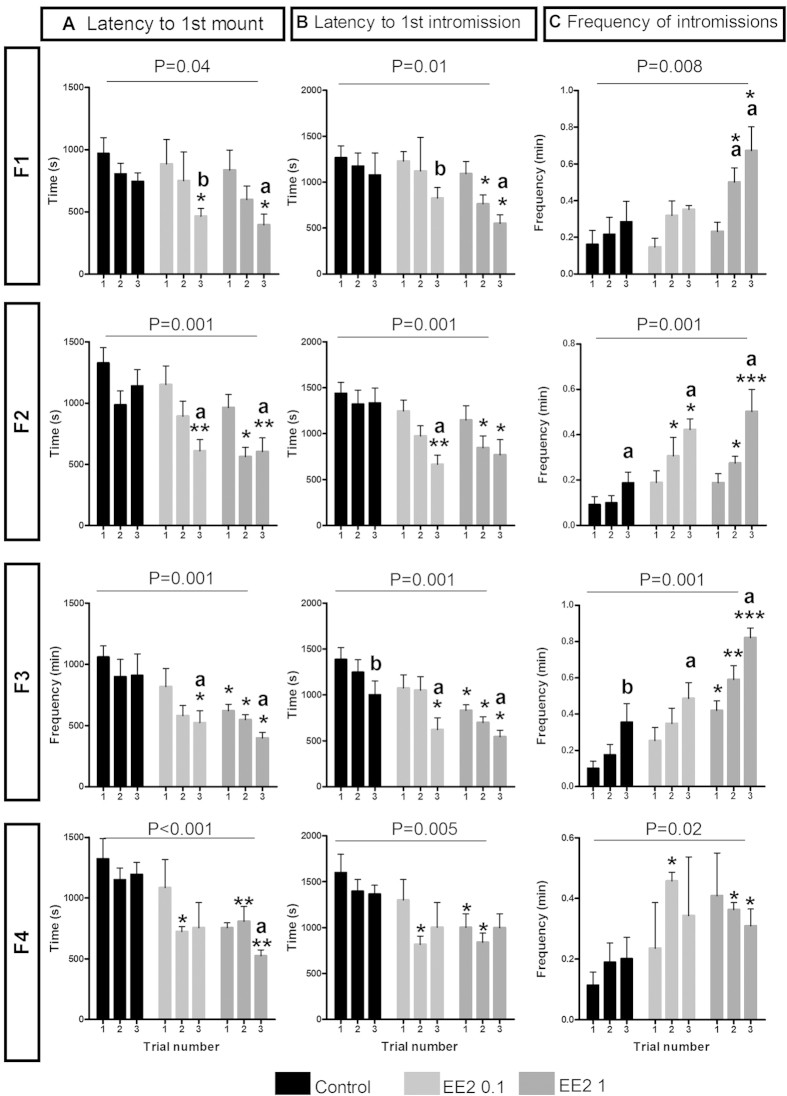
Developmental EE2 exposure affected male sexual behavior of adult F1 mice and their F2 to F4 progenies. Latencies *per* second to the first mount (**A**) and first intromission (**B**) and frequency of intromissions *per* minute (**C**) for each experimental group (Control, EE2 0.1 and EE2 1) (n = 3–5 litters for each group of each generation). Data are expressed as means ± SEM. *p-value < 0.05, **p-value < 0.01 and ***p-value < 0.001; EE2 0.1 or EE2 1 group *versus* Control group of the same trial test by Bonferroni *post-hoc* tests after two-way ANOVA. a: *p*-value < 0.05, aa: *p*-value < 0.01, aaa: *p*-value < 0.001, b: *p*-value = 0.07; significantly different from trial 1 using Friedman tests within each experimental group.

Sexual behavior was evaluated in males of the F1 generation directly exposed to EE2 and in their F2, F3 and F4 progenies. Three discrete components of male sexual behavior enabling sexual motivation and performance to be compared for each F1 to F4 male are presented in Fig. 1: latencies to first mount (A) and to first intromission (B), and frequency of intromissions (C) for each 30-minute test. The mean numbers of ano-genital investigations, attempted mounts and mounts without intromission are presented in Supplemental Material Table S1. As male sexual behavior improves with sexual experience, these parameters were evaluated over three tests. A two-way ANOVA was conducted with treatment and trial number as the two factors.

The F1 EE2-exposed males and their F2 to F4 progenies exhibited lower latencies of first mount and intromission, and higher frequencies of intromissions, with the effects being EE2 dose-dependent. The differences increased from the first to the third trial, demonstrating a greater improvement in sexual behavior in the EE2 0.1 and EE2 1 μg/kg/d groups than in the Control animals. These differences between males of the three experimental groups are statistically significant for the second and/or third trials. As an example, for the third trial, F1 male latencies to the first mount and first intromission were 765.3 ± 198.1 and 1078.9 ± 238.9 s for the Control males, and 465.4 ± 118.1 and 827.1 ± 114.0 s, and 395.5 ± 85.0 and 549.4 ± 96.63 s for the EE2 0.1 (*p*-value < 0.05) and EE2 1 (*p*-value < 0.05 and *p*-value < 0.001) groups respectively. In the Control group, the frequency of intromissions was 0.28 ± 0.0 intromission *per* minute, and 0.35 ± 0.0 and 0.67 ± 0.0 for the EE2 0.1 and EE2 1 groups (*p*-value < 0.01 and *p*-value < 0.001) respectively. In some cases, the differences between groups were even significant for the first trial; i.e. in F3 EE2 1 animals, the latency to first mount was 622.1 ± 51.3 s, compared to 1060.2 ± 89.4 s in Control males (*p*-value < 0.05).

### Male fertility

No differences in fertility were detected, since the relative number of litters and litter size did not vary (Supplemental Material Table S2). There was also no difference in sex ratio (number of males/number of females) between the three groups.

### EE2 developmental exposure and adult plasma testosterone concentrations

**Figure 2:**
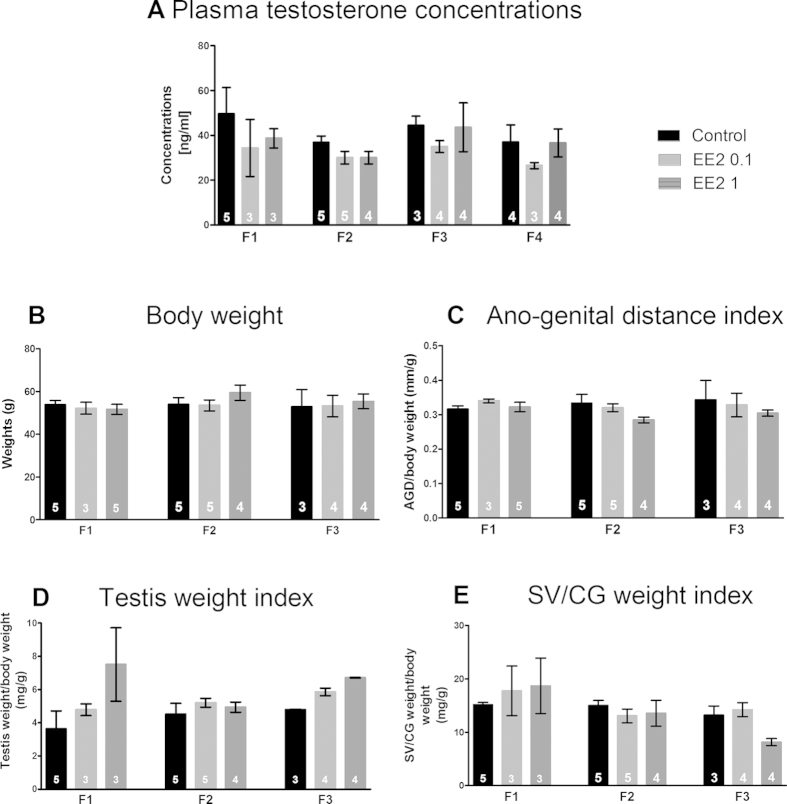
Developmental EE2 exposure did not alter reproductive physiology. EE2 exposure had no effect on plasma testosterone concentrations of F1 to F4 males (**A**), or on adult body weight (**B**), ano-genital distance (AGD) (**C**), testis weight (**D**) or seminal vesicles/coagulating glands (SV/CG) weight (**E**) of F1 to F3 males (n = 3–5 litters for each group of each generation) (Kruskal-wallis’ test). Each parameter measured was reported to the animal’s body weight to be expressed as an index of animal body weight. Since no significant alterations in reproductive tract morphometric parameters were detected, F4 males were not investigated for these analyses.

No significant difference was detected in the mean plasma testosterone concentrations between the males of the three experimental groups either for F1, or for F2, F3 or F4 generations (Fig. 2A).

### EE2 developmental exposure and genital tract in adults

There was no significant difference in animal weights between the three experimental groups (Fig. 2B). Figure 2C–E presents the mean indexes for ano-genital distances (AGD) (C), testes weights (D) and seminal vesicles/coagulating glands (SV/CG) weights (E) for F1 to F3 males. No significant difference was detected for any of these parameters. Nevertheless, although there was no significant change in mean VS/CG weights, recurrent abnormalities as right/left asymmetry in VS/CG of EE2 0.1 and EE2 1 males for the F1 and F2 generations were observed (see Supplemental Material Figure S2 for details). Moreover, testes sections counter-stained with eosin-haematoxylin were observed; no gross modification of testes histology was detected, but no quantification was performed (data not shown).

### Neuroanatomical studies

#### Effects of EE2 developmental exposure on hypothalamic GnRH neurons in adults

**Figure 3:**
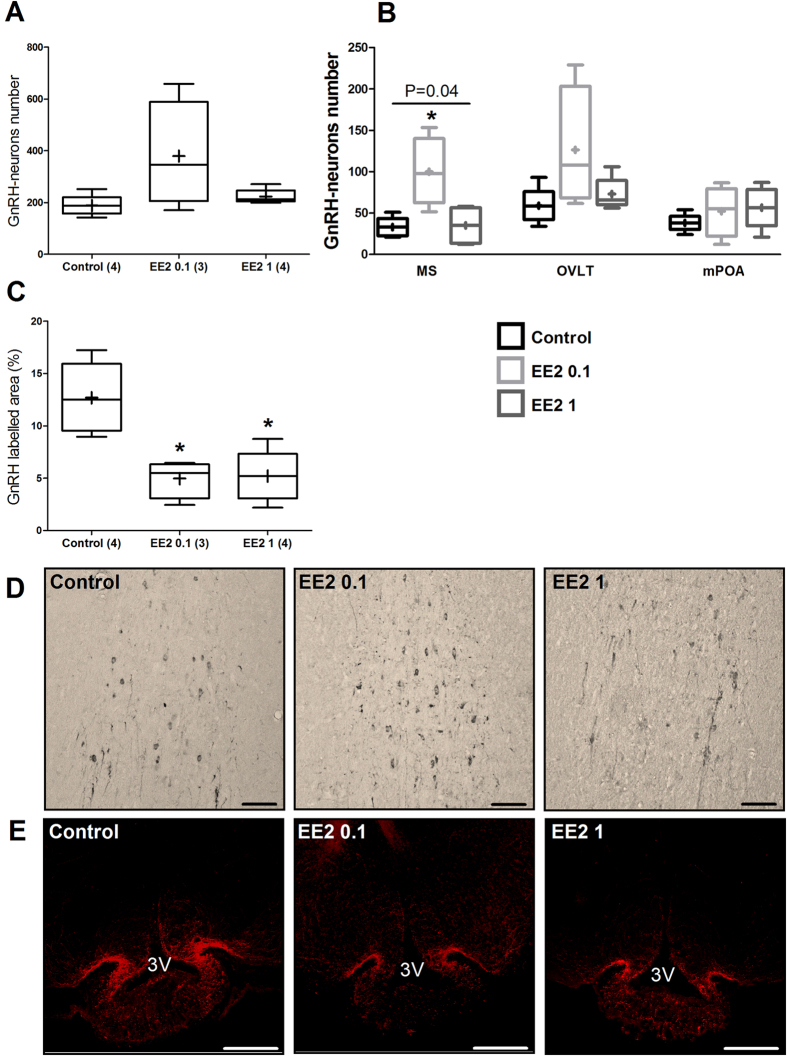
Developmental EE2 exposure disturbed GnRH neuron neuroanatomy in adult F1 male mice. (**A**) Tukey’s boxplots of total numbers of GnRH immunoreactive neurons in the hypothalamic POA for each experimental group. (**B**) Numbers and distribution of GnRH immunoreactive neurons according to the three neuroanatomical areas where cell bodies were counted: Median Septum (MS), *Organum Vasculosum Laminae Terminalis* (OVLT) and medial PreOptic Area (mPOA). (**C**) Box-plots of the mean GnRH labeled surface in three areas of the median eminence (ME; anterior, middle and posterior). The line in the middle of the box is plotted at the median and (+) is the mean. Numbers in brackets represent litter numbers. Kruskal-Wallis test with Dunn’s post-test with *p-value < 0.05. (**D**,**E**) Representative photographs of immunohistochemical labeling with DAD-Ni staining of GnRH neurons in the MS (**D**) and immunofluorescent terminal nerves in the ME (**E**) of Control, EE2 0.1 and EE2 1 males. Scale bars = 100 μm.

In F1 adult male mice the hypothalamic GnRH neuron perikarya were assessed, in terms of their numbers and neuroanatomical distribution (Fig. 3A,B) in the main areas where they are scattered (Median Septum (MS), *Organum Vasculosum Laminae Terminalis* (OVLT) and medial PreOptic Area (mPOA)), and their fiber density was recorded in the Median Eminence (ME), where most of the hypothalamic GnRH neurons project their axons (Fig. 3C).

No significant effect of EE2 treatment on the number of perikarya neurons was detected (Fig. 3A; Kruskal-Wallis test; *p*-value > 0.05). The neuroanatomical distribution of the GnRH neuron’ cell bodies through the MS, OVLT and mPOA revealed that in the Control group the majority of neurons were located in the OVLT (Fig. 3B). This distribution was not altered in the EE2 1 group but was significantly different in the MS of the EE2 0.1 group compared to the Control group (Kruskal-Wallis test with Dunn’s multiple comparison; *p*-value < 0.05) (Fig. 3B illustrated in Fig. 3D).

In males developmentally exposed to EE2, the average percentage of the GnRH labeled area in the ME was significantly lower in the EE2 0.1 (Kruskal-Wallis test; *p*-value = 0.02) and EE2 1 (Dunn’s multiple comparisons; *p*-values < 0.05) groups than in the Control group (Fig. 3C,E).

As F1 males exhibited no defect in fertility, the GnRH neuron network was not studied in their progeny.

#### EE2 developmental exposure and hypothalamic kisspeptin neurons in adults

**Figure 4:**
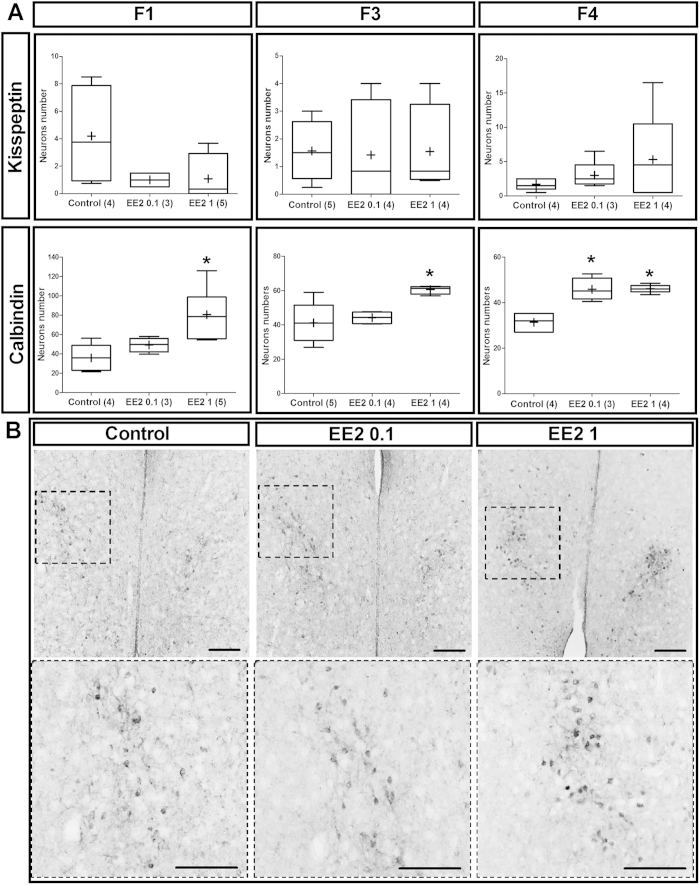
Unlike kisspeptin neurons, developmental EE2 exposure disturbed sexually dimorphic calbindin immunoreactive neurons in the medial PreOptic Area (mPOA). (**A**) Tukey’s boxplots of the number of kisspeptin and calbindin-immunoreactive neurons *per* brain section in mPOA of F1, F3 and F4 males. The line in the middle of the box is plotted at the median and (+) is the mean. Numbers in brackets represent litter numbers. Kruskal-Wallis test with Dunn’s post-test with *p-value < 0.05. (**B**) Representative anti-calbindin immunostaining in the mPOA showing the location of the sexually dimorphic calbindin population (NDS-POA). Images of the first panel present a low magnification giving an overview of the location of NDS-POA broadly delimited by the dotted squares magnified in the panel below. Scale bars = 100 μm.

The hypothalamic kisspeptin neurons are key regulators of GnRH neuron activity. In males, during development, these neurons are organized in the Periventricular preoptic (PVpo) nucleus by gonadal steroids^16^ rendering them potentially sensitive to xenoestrogens such as EE2. We therefore compared the numbers of immunoreactive kisspeptin neurons in the PVpo nuclei of Control and EE2-exposed males and no significant differences were observed for F1 generation animals or their F3 and F4 progeny (Fig. 4A).

#### Effects of EE2 developmental exposure on hypothalamic calbindin cells

Developmental exposure to EE2 significantly increased the number of calbindin immunoreactive (calb-ir) cells in the mPOA of F1 males of the EE2 1 group (80.5 ± 13.1) compared to the Control group (35.8 ± 8.0) (Kruskal-Wallis test; *p*-value = 0.04) (Fig. 4A), as illustrated in Fig. 4B. Similarly, in the mPOA of F3 males, EE2 increased the number of calb-ir neurons in the EE2 1 group (60.5 ± 1.2) compared to the Control group (41.2 ± 5.3) (Kruskal-Wallis test; *p*-value = 0.02). In F4 males, there were more calb-ir cells in both the EE2 0.1 (45.8 ± 2.5) and EE2 1 (46.1 ± 0.7) groups than in the Control group (31.3 ± 1.8) (Kruskal-Wallis test; *p*-value < 0.0001).

## Discussion

We demonstrated in the mouse a crucial impact of developmental exposure to both doses of EE2 (0.1 and 1 μg/kg/day) on male sexual behavior and calbindin neuronal population in the hypothalamic mPOA. EE2-exposed males expressed increased sexual behavior and had more calbindin cells in the mPOA. These effects were EE2 dose-dependent and were transmitted up to the F4 generation. Despite neuroanatomical modifications observed in the GnRH neuron population of F1 males under both doses of EE2 exposure, no major deleterious effects on reproductive physiology was detected in F1 adult males or their progenies.

The ability of an individual to recognize and adequately interact with a breeding partner constitutes a key step in reproductive success and species survival. The competence to respond to hormonal and social stimuli of breeding partners through appropriate behavior strongly depends on key developmental events during which neural circuits controlling these behaviors are correctly implemented^17^. We demonstrated that EE2-exposed animals exhibited a significant EE2 dose-dependent decrease in the latency to initiate sexual behavior and in the frequency of intromissions. This phenotype reflects an increase in the sexual behavior of EE2-exposed males. Differences in testosterone production of males from the three experimental groups could explain such increased male sexual behavior, but the plasma testosterone concentrations after hCG stimulation in EE2-exposed and Control males were unchanged. However, we did not evaluate whether the circulating levels of testosterone or the concentration of biologically active hormone in the bloodstream changed.

Beyond its major role in regulating adult male reproduction, testosterone plays a crucial part during development, both in the differentiation of the genital tract and of the brain^17^,^18^. The developmental sexual differentiation of specific brain areas such as the mPOA, involving local aromatization of testosterone into E2, is essential for the masculinization of neural networks by E2, producing permanent behavioral changes in adulthood. EE2 has a strong estrogenic effect and this could have induced a hypermasculinization of the brain leading to increased sexual behavior in EE2-exposed mice. To test this hypothesis of an effect of EE2 on the organization of brain neuroanatomy, independently of variations in testosterone levels, the sexual behavior of adult castrated males exposed to EE2 during development and treated with a same dose of exogenous testosterone should be investigated. This could illustrate an organizational effect of EE2 rather than a physiological influence due to the circulating level of testosterone.

The mPOA constitutes the primary neuroendocrine structure regulating male sexual behavior in different species^7^,^18^. Within the mPOA, the sexually dimorphic nucleus (SDN-POA) has been largely studied in the rat^19^ and other rodents^20^,^21^,^22^,^23^ and has been shown to be highly sensitive to aromatized androgens during the perinatal period in the male^24^,^25^,^26^. This structure is characterized by a cluster of calbindin cells whose number is greater in males than in females. It has recently been demonstrated that the mPOA might be a target for EDCs^27^. Our study found more calbindin immunoreactive cells in the mPOA of the F1 generation of EE2-exposed males and of their F3 and F4 offspring than in Control males, mainly in the EE2 1 group. This increase supports the hypothesis of a hypermasculinization effect of EE2 on brain structures involved in the control of male sexual behavior.

The modifications in male sexual behavior and calbindin cell numbers in the mPOA were observed in adulthood in F1 males exposed to EE2 during development. These changes, established early in life during the perinatal period, endure until adulthood. Moreover, these modifications were also observed in their progeny up to the F4 generation. Epigenetic processes constitute a mechanism by which endogenous and exogenous cues can control gene expression in the long-term. Notably, DNA methylation is associated with long-term DNA transcriptional repression. In the mouse, Nugent *et al.*^28^ demonstrated that masculinization of the POA and male sexual behavior lead to E2-mediated decreases in DNA methyltransferase enzymes. Moreover, in the rat, Anway *et al.*^29^ and Skinner & Guerrero-Bosagna^30^ showed that developmental exposure to the fungicide vinclozolin was able to alter mate preference and sperm epigenome, associated with epigenetic transgenerational inheritance for three generations. We can thus hypothesize that the increased male sexual behavior and the modifications in calbindin cells in the mPOA observed in our study over four generations may result from epigenetic processes, involving alterations in the sperm epigenome, which could be responsible for the transgenerational inheritance of the phenotypes. However these mechanisms remain to be characterized.

Hypothalamic GnRH neurons constitute the major center for controlling the HPG axis and thus the reproductive function^31^. In the current study, developmental exposure to EE2 did not modify the overall number of GnRH cell bodies in the POA, but induced a differential increase in immunoreactivity of GnRH neurons in the rostral region with more perikarya in the MS of the adult EE2 0.1 group males. This enhanced immunoreactivity may be due to a greater GnRH peptide content in perikarya leading to more neurons being detected by immunohistochemistry. However, we cannot exclude defects in the neuronal migration occurring between embryonic day 11 and 16 leading to misallocated neurons^32^,^15^. Furthermore, the dramatic decrease in the immunoreactivity of the GnRH terminal nerves in the ME in the EE2 0.1 and EE2 1groups could be explained either by a modification in GnRH peptide storage or transport from perikarya to terminal nerves, or by increased exocytotic release of peptide detected as neuropeptide depletion within the neurons. Clements *et al.*^33^ demonstrated in the rat that developmental exposure to dioxin induced changes in GnRH secretion in the ME linked to GnRH retention in perikarya. The major estrogen sensitive input onto GnRH neurons is the estrogen-sensitive kisspeptin neuronal network, but no effect on the dimorphic kisspeptin neuron population in the PVpo was observed in the present study. Altogether, despite the changes within the GnRH neuronal network, there was no major impact on reproductive physiology, fertility and genital tract anatomy.

One objective of this study was to determine whether oral exposure to low doses of EE2, below those tested in classical toxicology assays^34^ would disrupt neuroendocrine and behavioral outputs of male reproductive function. Two dose levels were tested; the lowest (0.1 μg/kg of body weight *per* day) corresponded to an environmental pollution occasionally found in aquatic environments^35^, and the highest (1 μk/kg of body weight *per* day) was within the range of a pharmacological dose^9^. An intake of EE2 at both 0.1 and 1 μkg/kg/day produced a dose-dependent effect on sexual behavior, calbindin cell number in the POA and GnRH fiber density in the ME. However, an effect on the number of GnRH soma was only observed at the lowest dose. This suggests that EE2 acts *via* different mechanisms depending on the target and the exposure time. It has been shown that EE2 has greater affinity to estrogen receptor ERα than ERβ^36^, but we know that hormone concentration or binding affinity does not necessarily predict the severity of the *in vivo* effect^37^,^38^,^39^. The POA highly expresses ERα which is strongly implicated in the sexual differentiation of neuroendocrine networks^40^. Moreover, the fact that ERβ-KO males show normal, strong sexual behavior^41^ implies that only ERα is important for sexual behavior. Together these data strongly suggest that the effects of EE2 on POA differentiation and sexual behavior are mediated by ERα rather than ERβ. However, during development, GnRH neurons express ERβ^42^, which has not been confirmed for ERα^43^, supporting the idea of a possible direct effect of EE2 on these neurons *via* ERβ. The absence of an effect at the highest EE2 dose could be related to differences in signaling pathways activated by different estrogen receptors at different EE2 concentrations. This hypothesis is in line with the well-established “low dose hypothesis” described in many EDCs studies and currently largely documented^44^. Several studies have reported that the effect of a low dose may be more potent than that of a higher dose for a specific parameter monitored towards a given endocrine active compound, particular regarding the risk assessment of reproductive parameters^45^. The nonlinear dose-response effect of endocrine-active molecules thus appears as the most significant and extensively peer-reviewed issue within the field of EDCs and it remains poorly understood (for review see refs 45,46).

## Conclusions

This study demonstrated that although developmental exposure to low doses of EE2 resulted in no overall deleterious effect on reproduction in adult male mice, sexual behavior and neuronal networks were impacted. These effects were transmitted to the progeny up to the F4 animals. Here we were able to highlight some effects which would not have been detected with toxicological regulatory studies^34^, since sexual behavior is not a parameter monitored *per se*. Such deep and transgenerational modifications of sexual behavior necessarily impact on individual and species fitness. Although care must obviously be taken when extrapolating from animal studies, it is possible to imagine the effect of such alterations induced by EE2 or other estrogenic EDCs on human health and the implied societal consequences. The relevance for effective health and safety of such new non-adverse, but biologically significant changes has to be seriously considered to understand the threats and to prevent risks to human health. Moreover, such long-term alterations in neuronal networks induced by developmental steroid perturbations may affect different social behaviors or even be involved in neurodegenerative diseases^47^. Demonstrating a significant elevation in progesterone and testosterone concentrations in amniotic fluids in the males with autism compared with controls, Baron-Cohen *et al.*^48^ suggest links between the prenatal hormone environment, sexually dimorphic behaviors and autism. This concern therefore needs to be considered by stakeholders and politicians to prevent long-term deleterious consequences.

## Methods

### Animals

Animals were obtained from fifteen pregnant Swiss mice (5 dams *per* experimental group; F0 generation) purchased from a commercial breeder (Charles Rivers, France). Mice were housed in individual standard cages during gestation and with their pups for lactation, and each male was housed with three brothers from weaning up to the beginning of the sexual behavior tests. Animals were given a free access to food and water under controlled temperature (22 °C) and photoperiod cycle (12 h light/12 h dark). All experiments were performed according to the European directive 2010/63/EU on the protection of animals used for scientific purposes and approved by an ethical committee for animal experimentation (CEEA Val-de-Loire, Tours, France, n°00302-01).

### Ethinylestradiol exposure

A stock solution of EE2 (Sigma Aldrich) was prepared in absolute ethanol (1 μg/mL) and dilutions to final doses of exposure were performed directly in drinking water. The daily dose was adjusted according to animals’ weights and water consumption. To estimate EE2 exposure, we previously evaluated the volume of drinking water ingested by a Swiss mouse per day (around 5 mL for an adult mouse). Animals were exposed to EE2 from embryonic day 10 until postnatal day (PND) 40 (F1 generation). This exposure period covered the whole developmental period, including *in utero* life from mid-gestation and the perinatal period until juvenile life, corresponding to the organizational window of neuroendocrine networks, and extended up to puberty, during which these neuroendocrine networks are activated^2^. The animals were divided into three groups. The first (EE2 0.1 group) was exposed to 0.1 μg of EE2 *per* kg body weight *per* day, a dose compatible with an exposure range found in highly polluted environments^35^. The second (EE2 1 group) was exposed to 1 μg of EE2 *per* kg body weight *per* day, corresponding to pharmacological exposure with a contraceptive pill or hormone replacement therapy^9^, and defined as the lowest observed adverse effect level (LOAEL) in humans^34^,^49^. The third (Control group) received vehicle. In order to avoid bias on animal growth due to litter size, each litter was reduced to 4 males and 4 females at PND4. Analyses were carried out on adult animals (from 8 weeks of age). The males tested for sexual behavior were different from the males included in the neuroanatomical studies.

Animals of the F2, F3 and F4 generations were obtained by crossing females and males within each experimental group. Thus F2, F3 and F4 males of the EE2 0.1 and EE2 1 groups originated from EE2-exposed parents, grand- and great-grand-parents respectively.

Each experimental group of each F1 to F4 generation included 12-20 males originating from 3 to 12 litters.

### Sexual behavior

Tests were performed in a dark room under red light. Naive males were individually housed for one week before the first test in a room devoid of the presence of any females. Each male of the F1, F2, F3 and F4 generations (n = 12–14 for each group of each generation obtained from 3 to 5 different litters *per* group) was tested three times, corresponding to the three trials, with a time interval of two weeks between trials. Males were tested in a cage containing fresh litter. After 10 min habituation an estrous female was introduced and the test lasted for 30 minutes. A mount involved a male placing its two anterior paws on either sides of the flank of the receptive female. Intromission was determined when the male carried out back and forth thrusting movements of the pelvic region. The numbers of ano-genital investigations and mount attempts, the latencies and the frequencies of mounts and intromissions were scored^41^.

Estrous females used as stimuli were ovariectomized under general anesthesia (xylazine/ketamine) and implanted with Silastic implants (Dow Corning) containing 50 μg of E2-benzoate (Sigma-Aldrich). Four hours before the tests, females were given a subcutaneous injection of 1 mg of progesterone (Sigma-Aldrich) diluted in 100 μL of oil to induce receptivity.

### Fertility study

Male fertility was assessed at the end of the behavioral analyses. A male mouse was housed with a female for 1 to 14 days until a vaginal plug was observed. The number of litters and pups *per* litter were recorded for each experimental group of males.

### Plasma testosterone concentrations

Two hours before being euthanized, males of the F1 to F4 generations were injected intraperitoneally with 15 UI of human chorionic gonadotropin (hCG) (Intervet, France) diluted in physiological serum^50^. Blood was collected at death, plasma separated and stored at −20 °C until testosterone assays. Plasma testosterone concentrations were evaluated through a RIA using ^3^H testosterone as previously described^51^. The sensitivity of the assay was 0.115 ng/mL and the intra-assay coefficient of variation was 7%.

### Genital tract analysis

When F1, F2 and F3 animals were euthanized, each male was weighted, the ano-genital distance (AGD) was measured, and testes, seminal vesicles and coagulating glands (SV/CG) were weighted.

### Immunohistochemistry

Brains were collected according to the protocol described in Geller *et al.*^52^. After embedding in TissuTek^®^, brains were cut with a cryostat (Leica) into 20 μm coronal slices, stored at -20 °C until immunohistochemistry. Slices were processed for immunostaining with rabbit polyclonal anti-GnRH antibody (1:3000; ref. 52), sheep anti-kisspeptin antibody (1:2000; ref. 53) or mouse monoclonal anti-calbindin antibody (1:5000; C8948; clone CB-955; Sigma-Aldrich). GnRH immunostaining was performed in the POA and ME of F1 animals, and kisspeptin and calbindin immunostainings were performed respectively in the PVpo nucleus and mPOA of F1, F3 and F4 animals. Details of all the procedures are provided in Supplemental Material Part1. No immunohistochemical analysis could be performed on F2 animals because of technical troubles.

### Analysis and quantification of immunohistochemical studies

GnRH cell numbers were analyzed under an optical microscope at 20X magnification in three neuroanatomical regions: Median Septum (MS), *Organum Vasculosum Laminae Terminalis* (OVLT) and medial PreOptic Area (mPOA). In each region, GnRH immunoreactive cell bodies were counted on 10 consecutive coronal sections. The mean numbers of GnRH immunoreactive neurons were compared between the experimental groups both overall by summing the numbers determined in the three regions and within each neuroanatomical region.

Analysis of GnRH terminal nerves in the ME was performed using epifluorescence microscopic images computerized with Mercator Software (Explora Nova, La Rochelle, France). Three coronal slices corresponding to the anterior, middle and posterior parts of the ME were selected according to Paxinos & Franklin’s brain atlas^54^ (Supplemental Material Figure S1). The proportion of the area of GnRH fibers which were immunoreactive was quantified in 10.000 μm^2^ taken from and centered on the ME (20X magnification). Results are presented as the distribution of mean labeled surface area *per* litter (1–2 animals *per* litter) for each group.

Kisspeptin neurons were counted on confocal images (LSM 700, 20X magnification) with Zen^®^ software. For each animal, two brain coronal slices corresponding to the PVpo were analyzed. All the labeled neurons distributed around the third ventricle were counted and a mean number of neurons *per s*ection and *per* animal was obtained. Counting of calbindin positive cells in the mPOA was carried out on images taken at 20X magnification according to a method adapted from ref. 26. Details are described in Supplemental Material Part2. The values represent the mean numbers of cells counted on one side of the third ventricle.

### Statistical analyses

Results are presented as average values *per* litter for each parameter monitored and obtained from values of animals from the same litter. For each litter one to two animals were used for neuroanatomical studies and two to four animals were analyzed for behavior. Statistical analyses were performed with GraphPad Prism5^®^ software (GraphPad Software, San Diego, CA). Data were compared using a non-parametric Kruskal Wallis test with a Dunn’s post-test. Behavioral parameters were analyzed using a two-way ANOVA test with a Bonferroni post-test. A Friedman test was used for intragroup comparisons (trial number effect). A Chi-square test was used to compare frequencies. Differences were considered significant for *p*-value < 0.05. Data presented in the histograms are means ± Standard Error of the Mean (SEM).

## Additional Information

**How to cite this article**: Derouiche, L. *et al.* Developmental exposure to Ethinylestradiol affects transgenerationally sexual behavior and neuroendocrine networks in male mice. *Sci. Rep.* **5**, 17457; doi: 10.1038/srep17457 (2015).

## Supplementary Material

Supplementary Information
