# Supplementary material for: Developmental exposure to Ethinylestradiol affects transgenerationally sexual behavior and neuroendocrine networks in male mice
Source: Sci Rep. 2015 Dec 7;5:17457. doi: 10.1038/srep17457 (PMC4671018; doi:10.1038/srep17457)
Supplement: Supplementary Information [file srep17457-s1.doc]

**Supplementary Information**

**Developmental exposure to Ethinylestradiol affects transgenerationally sexual behavior and neuroendocrine networks in male mice**

Lyes DEROUICHE^1^

Matthieu KELLER^1^

Anne Hélène DUITTOZ^1^

Delphine PILLON^1*^

^1^ PRC, UMR 7247 INRA / CNRS / Université François-Rabelais de Tours / IFCE, Nouzilly, France

* Corresponding author

**Table of Contents:**

**- Part 1: Immunohistochemistry.**

**- Part 2: Analysis and quantification for the calbindin immunohistochemical study.**

**- Figure S1: Analysis and quantification of the GnRH immunohistochemical study in the median eminence.**

**- Figure S2: Occurrence of abnormalities in seminal vesicles and coagulating glands (SV/CG)**.

**- Table S1: Male sexual behavior in F1 to F4 animals.**

**- Table S2: Male fertility in F1 to F3 animals.**

**Part 1: Immunohistochemistry.**

For immunostaining of GnRH, kisspeptin and calbindin, slices were immersed in PBS containing 0.3 Triton X100 (PBST) and 1% H_2_O_2_ for 15 minutes. The sections were blocked for 1 hour in 10% normal goat serum in PBST (PBST-NGS), and then incubated overnight at 4°C with rabbit polyclonal anti-GnRH antibody (1:3000; ref^52^), sheep anti-kisspeptin antibody (1:2000; ref^53^) or mouse monoclonal anti-calbindin antibody (1:5000; C8948; clone CB-955; Sigma-Aldrich). GnRH and calbindin immunostaining in the POA was carried out over a 2-hour period at room temperature (RT) using biotinylated goat anti-rabbit (1:500; Vector laboratories) or anti-mouse (1:250) immunoglobulin antibodies respectively. Bound antibodies were visualized after incubating for 1 hour with the ABC horseradish peroxidase complex (1:600; Vector Burlingame, CA, USA kit Vectastain Elite (PK6100)). The signal was revealed with 3’3-diaminobenzidine and 0.02 % H_2_O_2_ (Sigma-Aldrich).

For immunostaining of GnRH in the ME and kisspeptin in the PVpo, GnRH and kisspeptin immunofluorescence labeling was carried out over a 2-hour period at RT using Alexa 546-conjugated goat anti-rabbit or donkey anti-sheep IgG second antibody (1:1000; Molecular Probes) respectively. After three rinses in PBS, nuclei were counterstained with DAPI (1:1000) incubating for one minute. Slides were rinsed in water and coverslipped with Fluoromount-G (Southern Biotechnology, Birmingham, AL, USA) before being stored at 4°C in the dark.

**Part 2: Analysis and quantification for the calbindin immunohistochemical study.**

For each animal, four sections were selected in the area of interest, two adjacent sections and two other sections separated by 100 µm from the first two. The area counted was delimited by a 200 µm^2^ frame, placed laterally at a distance of 200 µm from the wall of the third ventricle and 600 µm from the optic chiasma. On ImageJ® software (NIH), each image was binarized and the cell bodies were pointed using the cell counter plugin.

**Figure S1:** **Analysis and quantification of the GnRH immunohistochemical study in the median eminence.**

Drawings from *The Mouse Brain in Stereotaxic Coordinates* (from ref^54^) illustrating the areas analyzed from the anterior **(A)**, middle **(B)** and posterior **(C)** median eminence (ME). **(D, E, F)** GnRH labeling by immunofluorescence from Control individual in the three areas corresponding respectively to the boxes above. Scale bar = 100 µm.

**Figure S2: Occurrence of abnormalities in seminal vesicles and coagulating glands (SV/CG)**. Abnormality was defined as asymmetrical SV/CG exhibiting remarkable differences in size between right and left SV/CG. **(A)** Histograms of the percentage of asymmetrical SV/CG in F1 and F2 males. No asymmetrical SV/CG were observed in F3. ***: *p*-value<0.001: Fisher’s exact test, statistically different from Control. The numbers at the bottom of the histograms represent the number of animals examined. **(B)** Representative photomicrographs of normal and asymmetrical SV/CG. No abnormality was ever observed in males of the Control group. Scale bar = 5 mm.

**Table S1: Male sexual behavior in F1 to F4 animals.**

The means of the number of ano-genital investigations, attempted mounts and mounts without intromission for each experimental group (Control, EE2 0.1 and EE2 1) of the F1, F2, F3 and F4 generations, throughout the three tests (trials 1 to 3) (n=3-5 litters for each group of each generation) were calculated. Data are expressed as means number of the event ± SEM for each trial and for the three groups. Two-ways ANOVA with Bonferroni post-test: *: *p*-value<0.05, **: *p*-value<0.01, ***: *p*-value<0.001.

**Table S2: Male fertility in F1 to F3 animals.**

The relative number of litters (percentages of mated females farrowed) and the sex-ratio (number of males / number of females) for each experimental group (Control, EE2 0.1 and EE2 1) of the F1, F2 and F3 generations were calculated. The litter size is also indicated. (Kruskal-Wallis test, *p*-value>0.05) (n=5-12 litters for each group of each generation).

**Table S1**

|  | **Ano-genital investigation (AGI)** | | | **Attempted mounts (AM)** | | | **Mounts without intromission (MWI)** | | |
| --- | --- | --- | --- | --- | --- | --- | --- | --- | --- |
|  | **Trial1** | **Trial2** | **Trial3** | **Trial1** | **Trial2** | **Trial3** | **Trial1** | **Trial2** | **Trial3** |
| Control | 16.62 ± 1.14 | 19.14 ± 1.14 | 17.53 ± 1.56 | 5.68 ± 1.28 | 6.06 ± 1.72 | 5.60 ± 2.62 | 5.35 ± 1.2 | 4.10 ± 1.67 | 8.41 ± 1.66 |
| EE2 0.1 | 13.22± 86 | 14.86 ± 4.86 | 15.16 ± 4.73 | 7.34 ± 2.90 | 8.41 ± 2.45 | 6.41± 0.71 | 5.55± 1.14 | 5.5 ± 2.50 | 11.25 ± 1.42 |
| EE2 1 | 14.53 ± 2.52 | 14.86 ± 1.36 | 16.48 ± 2.83 | 6.76 ± 1.76 | 7.50 ± 2.05 | 6.73 ± 1.34 | 5.90 ± 1.47 | 8.88 ± 1.17 | 9.21 ± 1.72 |
| Control | 21.20 ± 2.87 | 24.33 ± 2.89 | 26.16 ± 3.98 | 2.69 ± 0.69 | 2.96 ± 0.45 | 1.68 ± 0.66 | 1.80 ± 0.47 | 4.33 ± 0.97 | 2.68 ± 12.12 |
| EE2 0.1 | 24.05 ± 3.96 | 15.036± 4.17 | 18.62± 1.89 | 1.50 ± 0.99 | 3.26 ± 0.62 | 2.6 ± 0.78 | 1.72 ± 0.67 | 3.13 ± 1.34 | 10.61± 2.27*** |
| EE2 1 | 23.72 ± 4.37 | 25.91 ± 4.02 | 28.31 ± 3.15 | 3.63 ± 0.59 | 3.55 ± 0.34 | 3.12 ± 0.81 | 3.43 ± 0.73 | 3.81 ± 1.08 | 6.37 ± 1.37* |
| Control | 34.52 ± 2.99 | 24.06 ± 2.23 | 30.06 ± 1.44 | 4.45± 0.20 | 3.45 ± 1.01 | 2.80 ± 0.45 | 3.62 ± 0.59 | 5.23 ± 1.31 | 5.90 ± 1.42 |
| EE2 0.1 | 28.00± 2.72 | 23.75 ± 2.96 | 22.60 ± 1.64 | 3.40 ± 0.67 | 3.37 ± 0.93 | 3.00 ± 0.76 | 5.00 ± 1.01 | 8.25± 1.31 | 6.35 ± 1.50 |
| EE2 1 | 29.33 ± 2.04 | 24.670± 4.45 | 25.33 ± 2.09 | 3.25 ± 0.47 | 2.79 ± 1.18 | 3.08 ± 0.51 | 4.54 ± 0.46 | 6.14 ± 1.56 | 8.45 ± 1.51 |
| Control | 29.125 ± 1.54 | 32.18 ± 1.13 | 17.93 ± 1.55 | 2.12 ± 0.41 | 1.43 ± 0.20 | 2.87 ± 0.30 | 2.12 ± 0.37 | 4.31 ± 0.78 | 5.00 ± 1.16 |
| EE2 0.1 | 20.58 ± 3.41 | 19.91 ± 0.95 | 15.66 ± 2.22 | 3.08 ± 0.11 | 2 ± 0.34 | 2.36 ± 0.27 | 3.50 ± 0.78 | 4.16 ± 0.52 | 3.77 ± 0.32 |
| EE2 1 | 32.31 ± 2.69 | 29.54± 1.27 | 21.12 ± 2.27 | 3.41 ± 0.08 | 1.66 ± 0.33 | 5;04 ± 0.88 | 5.10 ± 0.75 | 5.72 ± 1.72 | 7.77 ± 1.29 |

**Table S**2

|  |  | **Relative number of litters** (%) | **Litter size** | **Sex ratio** |
| --- | --- | --- | --- | --- |
| F1 | Control | 100 | 11.3 ± 0.8 | 0.98 ± 0.20 |
|  | EE2 0.1 | 100 | 12.4 ± 1.1 | 1.22 ± 0.17 |
|  | EE2 1 | 100 | 10.5 ± 1.3 | 1.17 ± 0.27 |
| F2 | Control | 100 | 8.2 ± 1.9 | 0.56 ± 0.13 |
|  | EE2 0.1 | 83 | 8.0 ± 2.0 | 0.79 ± 0.19 |
|  | EE2 1 | 67 | 11.0 ± 1.7 | 0.61 ± 0.13 |
| F3 | Control | 80 | 8.4 ± 0.9 | 0.93 ± 0.16 |
|  | EE2 0.1 | 60 | 9.1 ± 1.2 | 1.12 ± 0.14 |
|  | EE2 1 | 80 | 8.6 ± 1.0 | 0.82 ± 0.17 |

**Figure S1**

**
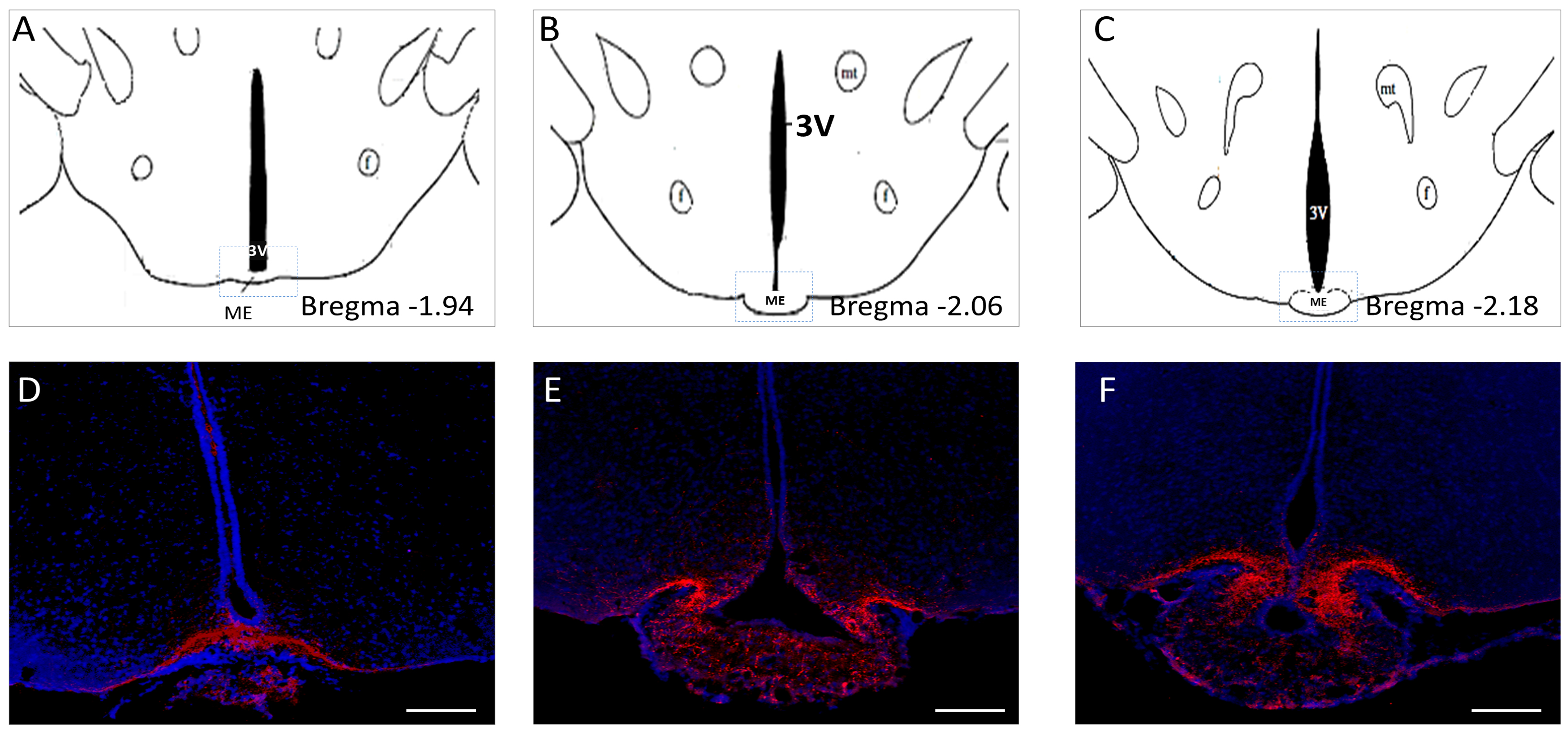
**

**Figure S2**

**
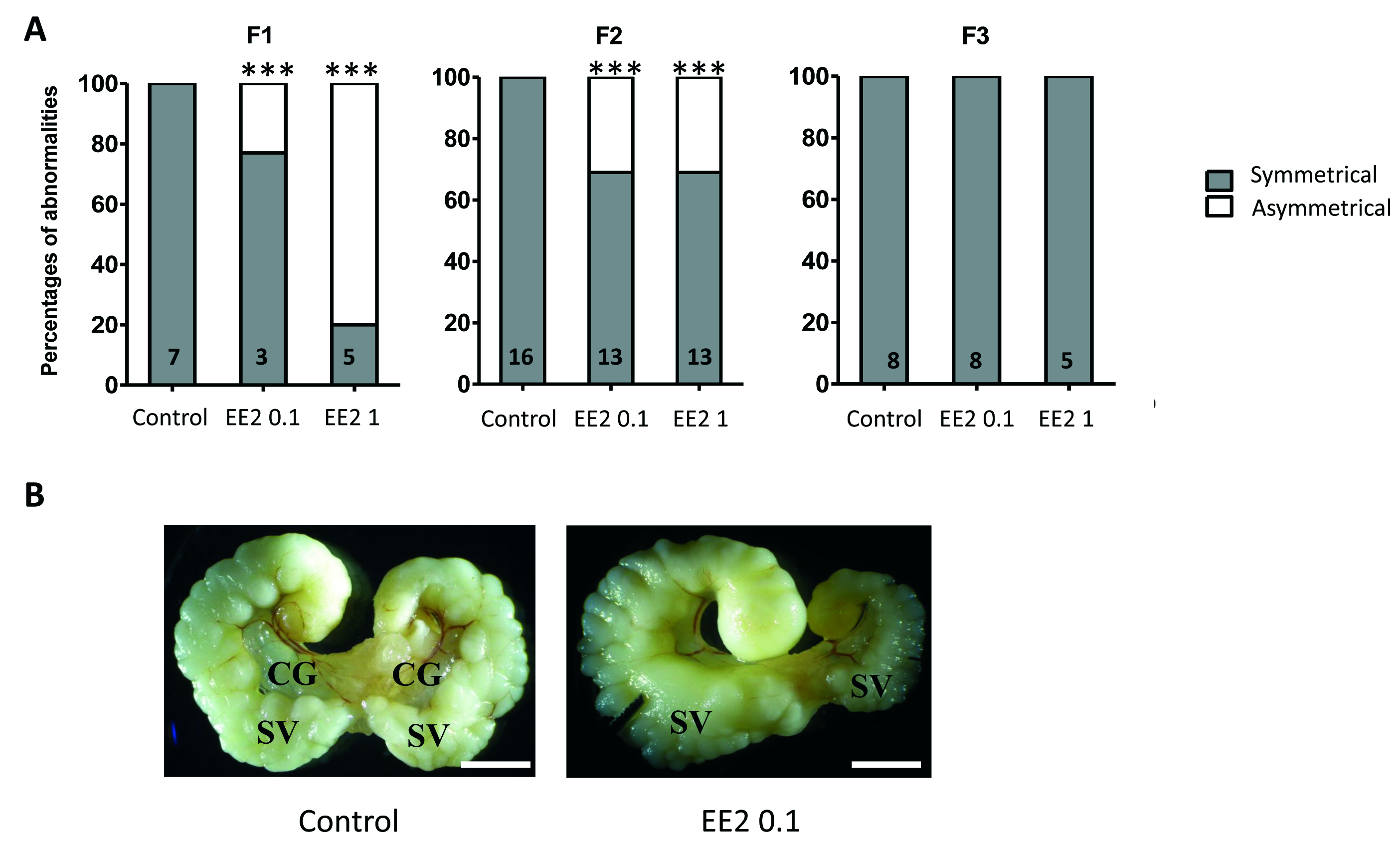
**
